# The Parasitic Dinoflagellates *Blastodinium* spp. Inhabiting the Gut of Marine, Planktonic Copepods: Morphology, Ecology, and Unrecognized Species Diversity

**DOI:** 10.3389/fmicb.2012.00305

**Published:** 2012-08-28

**Authors:** Alf Skovgaard, Sergey A. Karpov, Laure Guillou

**Affiliations:** ^1^Laboratory of Aquatic Pathobiology, Department of Veterinary Disease Biology, University of CopenhagenFrederiksberg, Denmark; ^2^Zoological Institute of the Russian Academy of SciencesSt. Petersburg, Russia; ^3^St. Petersburg State UniversitySt. Petersburg, Russia; ^4^Station Biologique de Roscoff, Université Pierre et Marie Curie – Paris 6Roscoff, France; ^5^Laboratoire Adaptation et Diversité en Milieu Marin, CNRS, UMR 7144Roscoff, France

**Keywords:** *Blastodinium*, copepod, parasite, symbiont, plankton, ultrastructure, phylogeny

## Abstract

*Blastodinium* is a genus of dinoflagellates that live as parasites in the gut of marine, planktonic copepods in the World’s oceans and coastal waters. The taxonomy, phylogeny, and physiology of the genus have only been explored to a limited degree and, based on recent investigations, we hypothesize that the morphological and genetic diversity within this genus may be considerably larger than presently recognized. To address these issues, we obtained 18S rDNA and ITS gene sequences for *Blastodinium* specimens of different geographical origins, including representatives of the type species. This genetic information was in some cases complemented with new morphological, ultrastructural, physiological, and ecological data. Because most current knowledge about *Blastodinium* and its effects on copepod hosts stem from publications more than half a century old, we here summarize and discuss the existing knowledge in relation to the new data generated. Most *Blastodinium* species possess functional chloroplasts, but the parasitic stage, the trophocyte, has etioplasts and probably a limited photosynthetic activity. Sporocytes and swarmer cells have well-developed plastids and plausibly acquire part of their organic carbon needs through photosynthesis. A few species are nearly colorless with no functional chloroplasts. The photosynthetic species are almost exclusively found in warm, oligotrophic waters, indicating a life strategy that may benefit from copepods as microhabitats for acquiring nutrients in a nutrient-limited environment. As reported in the literature, monophyly of the genus is moderately supported, but the three main groups proposed by Chatton in 1920 are consistent with molecular data. However, we demonstrate an important genetic diversity within the genus and provide evidences for new groups and the presence of cryptic species. Finally, we discuss the current knowledge on the occurrence of *Blastodinium* spp. and their potential impact on natural copepod populations.

## Introduction

**Figure 1:**
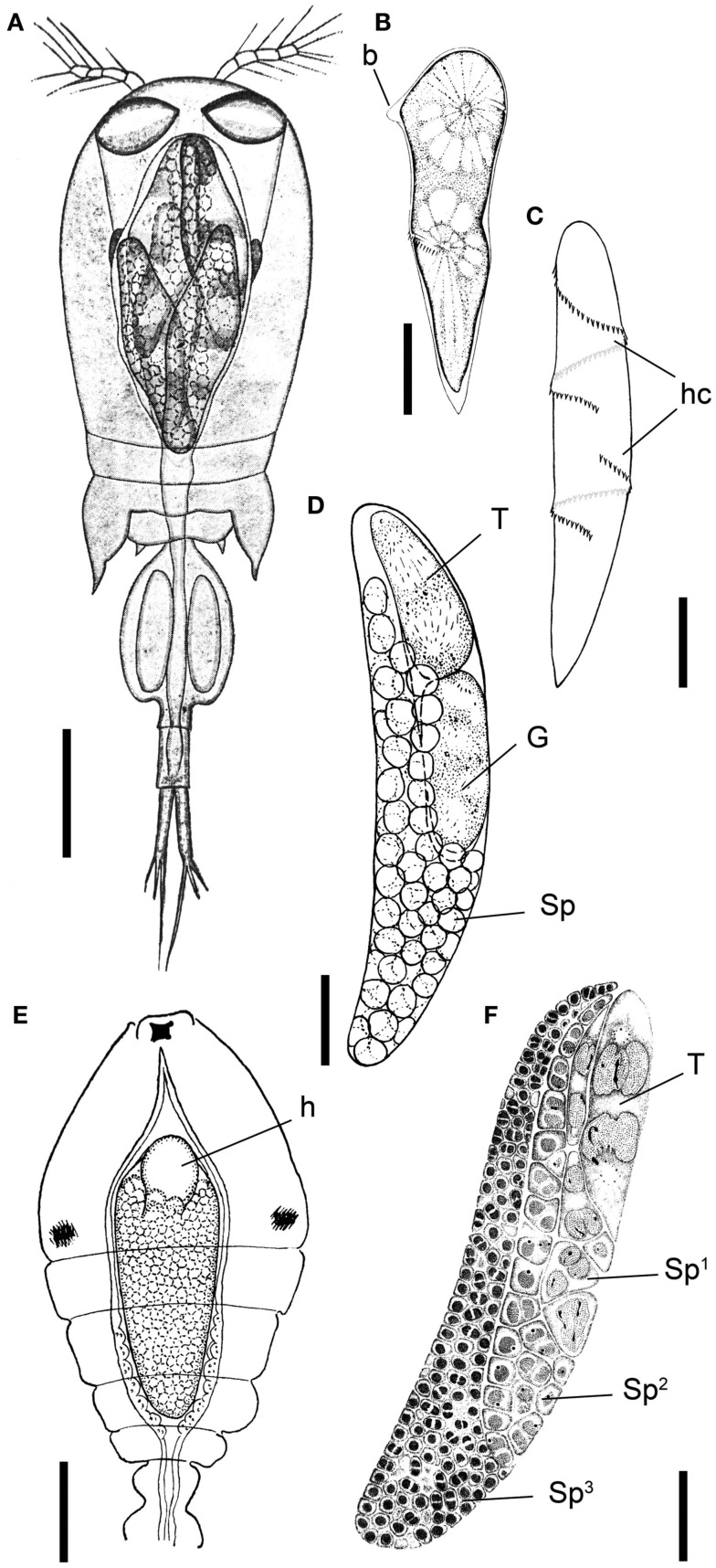
***Blastodinium* spp. (A)** Several *B. navicula* inside the gut of the copepod *Corycaeus giesbrechti*. **(B)** A young trophocyte of *B. pruvoti* with anterior beak (b). **(C)** Early stage *B. spinulosum* with helicoidal crests (hc). **(D)** *B. pruvoti* showing trophycyte (T), gonocyte (G), and sporocytes (Sp). **(E)** *B. oviforme* with hilum (h) in *Oithona nana*. **(F)** *B. pruvoti* with three progressive layers of sporocytes (Sp^1^–Sp^3^). Adapted from Chatton (1920). **(A,E)** Scale bars = 100 μm. **(B–F)** Scale bars = 50 μm.

The typical dinoflagellate is a motile, bi-flagellated protist, and species of the group may be found in both marine and fresh waters. Roughly half of all dinoflagellates are photosynthetic and half are heterotrophic (Gaines and Elbrächter, 1987). In addition, it has been estimated that approximately 7% of the dinoflagellates have parasitic life strategies (Drebes, 1984), infecting other protists, cnidarians, crustaceans, fishes, etc. (Coats, 1999) and some of these parasites can be severe pathogens for wild and farmed aquatic organisms. A key morphological feature of the dinoflagellates is their nucleus, the dinokaryon, which differs from the typical eukaryote nucleus by having permanent condensed chromosomes and by lacking histones. However, some of the parasitic dinoflagellates deviate from this typical morphology. In the dinoflagellate order Syndiniales a dinokaryon is never present, and those species that have traditionally been referred to the order Blastodiniales are believed to have a dinokaryon only in some parts of their live cycles. *Blastodinium* is a genus of dinoflagellates that appears atypical in several aspects. The parasitic stage of *Blastodinium* exists exclusively inside the gut of marine free-living copepods, where it occupies the lumen of the intestinal tract (Figure 1A). This parasitic stage is multicellular. It consists of several hundred non-flagellated cells, and can reach a length of more than 1 mm. The dispersal stage, the dinospore, of *Blastodinium* has the morphology of a typical dinoflagellate.

The greater part of our existing knowledge on *Blastodinium* leads back to the immense work done by the French protozoologist Édouard Chatton (Soyer-Gobillard, 2006) in the first half of the twentieth century, and his 1920 monograph (Chatton, 1920) on the parasitic dinoflagellates is still the primary landmark of several dinoflagellate genera and in particular of the genus *Blastodinium*. In addition to discovering and describing the genus in 1906, Chatton also described most known *Blastodinium* species in succeeding publications (Table 1). Since then, only three new species of *Blastodinium* have been described (Sewell, 1951; Skovgaard and Salomonsen, 2009), but recent investigations have shown that the genetic diversity within the genus is high, suggesting a larger number of unknown species (Coats et al., 2008; Skovgaard and Salomonsen, 2009; Alves-de-Souza et al., 2011). Investigations of the biology and impact of *Blastodinium* spp. are remarkably rare considering the large amount of research that is continuously being carried out on the biology and ecology of marine copepods. In this paper we update the current knowledge on the biology, phylogeny, and morphology of *Blastodinium* spp. and the current reports of distribution and host range of *Blastodinium* spp. are summarized. The established data is supplemented with new observations on morphology, ultrastructure, molecular phylogeny, and photosynthetic potential of *Blastodinium* spp. Due to the photosynthetic capability of *Blastodinium* and the apparently weak pathology associated with the infection, it may be a matter of definition whether members of the genus should be categorized as parasites or symbionts. We here adopt the term parasite because some harmful effect has been documented in association with hosting a *Blastodinium*.

**Table 1:** **Recognized species of *Blastodinium* and their reported copepod hosts**.

Parasite (Author, year)	Hosts	Reference
***Spinulosum* group**	**Calanoida, Cyclopoida, Poecilostomatoida**	
*B. spinulosum* (Chatton, 1908)	*Acrocalanus gracilis* Giesbrecht	2
	*Clausocalanus arcuicornis* Dana	1
	*C. furcatus* Brady	1
	*C. farrani* Sewell	2
	*Paracalanus aculeatus* Giesbrecht	2
	*P. denudatus* Sewell	2
	*P. parvus* Claus	1
*B. pruvoti* (Chatton, 1906)	*Clausocalanus arcuicornis*	1
	*C. furcatus*	1
	*Nannocalanus minor* Claus	2
	*Paracalanus parvus*	2
*B. crassum* (Chatton, 1908)	*Paracalanus parvus*	1
	*P. nanus* Sars	2^+^
	*Calocalanus styliremis* Giesbrecht	1
	*Clausocalanus arcuicornis*	1
	*C. furcatus*	1
	*P. aculeatus*	2^+^
*B. inornatum* (Chatton, 1920)	*Clausocalanus arcuicornis*	1
	*C. furcatus*	1
	*Paracalanus parvus*	1
*B. oviforme* (Chatton, 1912)	*Corycaeus crassiusculus* Dana	2
	*C. speciosus* Dana	2
	*Farranula gibbula* Giesbrecht	2
	*Oithona similis* Claus	1
	*O. nana* Giesbrecht	1
	*O. plumifera* Baird	1
	*Oncaea media* Giesbrecht	2
	*O. venusta* Philippi	2
	*Triconia conifera* Giesbrecht	2
***Contortum* group**	**Calanoida**	
*B. contortum* (Chatton, 1908)	*Acartia clausi* Giesbrecht	1
	*Acrocalanus gracilis*	2
	*Calocalanus styliremis*	1
	*Clausocalanus arcuicornis*	1
	*C. furcatus*	1
	*C. lividus* Frost & Fleminger	3?
	*Cosmocalanus darwini* Lubbock	2
	*Eucheata indica* Wolfenden	2
	*Nannocalanus minor*	2
	*Paracalanus aculeatus*	2
	*P. denudatus*	2
	*P. parvus*	1
	*Subeucalanus pileatus* Giesbrecht	2
	*S. subtenuis* Giesbrecht	5?
	*Temora stylifera* Dana	Figures 11E,F
*B. hyalinum* (Chatton, 1929)	*Acartia clausi*	4/1
	*Calanus finmarchicus* Gunnerus	4/1
	*Centropages* sp.	1
	*Clausocalanus arcuicornis*	4/1
	*C. furcatus*	1
	*Paracalanus aculeatus*	1
	*P. denudatus*	2
	*P. parvus*	2
	*Paracalanus* sp.	1
	*Paraeuchaeta antarctica* Giesbrecht	4/1
	*Pseudocalanus elongatus* Boeck	6?
	*Pseudocalanus* sp.	2
*B. apsteini* (Sewell, 1951)	*Clausocalanus arcuicornis*	1/2
	*C. furcatus*	2
	*Paracalanus aculeatus*	2
*B. chattoni* (Sewell, 1951)	*Clausocalanus arcuicornis*	2
	*C. furcatus*	2
	*Cosmocalanus darwini*	2
	*Eucheata indica* Wolfenden	2
	*Nannocalanus minor*	2
	*Paracalanus aculeatus*	2
	*P. denudatus*	2
	*P. parvus*	2
***Mangini* group**	**Poecilostomatoida, Calanoida**	
*B. mangini* (Chatton, 1908)	*Farranula gibbula* Giesbrecht	2^#^
	*F. rostrata* Claus	1*
	*Oncaea media* Giesbrecht	2^#^
	*O. venusta* Philippi	2^#^
	*Oncaea* cf. s*cottodicarloi* Heron & Bradford-Grieve	7^#^
	*Triconia conifera* Giesbrecht	2^#^
*B. mangini* var. *oncaea* (Chatton, 1912)	*F. rostrata*	1
	*O. media*	1*
	*Triconia minuta* Giesbrecht	1
*B. navicula* (Chatton, 1912)	*Corycaeus giesbrechti* Dahl	1
	*O. venusta*	2*
*B. elongatum* (Chatton, 1912)	*Centropages* sp.	1
	*Scolecithrix bradyi* Giesbrecht	1
*B. galatheanum* Skovgaard	*Acartia negligens* Dana	8
	*Acartia* sp.	8
***Blastodinium* spp**.		
*Blastodinium* sp. α	*Temora stylifera*	1
*Blastodinium* sp. β	*Clausocalanus furcatus*	1
*Blastodinium* sp. γ	*C. arcuicornis*	1
	*Paracalanus parvus*	1
*Blastodinium* sp. δ	*Corycaeus giesbrechti*	1
*Blastodinium* spp.	*Acrocalanus longicornis* Giesbrecht	5
	*Aetidius giesbrechti* Cleve	3
	*Centropages typicus* Krøyer	3
	*Clausocalanus furcatus*	3
	*C. parapergens* Frost & Fleminger	10
	*Corycaeus flaccus* Giesbrecht	3
	*C. typicus* Krøyer	3
	*Euchaeta rimana* Bradford	5
	*Farranula carinata* Giesbrecht	9
	*Nannocalanus minor*	3
	*Neocalanus robustior* Giesbrecht	5
	*Pareucalanus sewelli* Fleminger	5
	*Pleuromamma gracilis* Claus	3
*Blastodinium* sp. I	*Euchaeta* sp.	Figures 6A,B
*Blastodinium* sp. II	*Euchaeta* sp.	Figures 6C,D

*The genera *Oncaea* and *Triconia* have been thoroughly revised since the work of Chatton (1920) and Sewell (1951) signifying that these early observations probably included several at that time unrecognized host species.

*^+^No distinction was made between *B. crassum* and B*. inornatum**.

*^#^No distinction was made between *B. mangini* and *B. mangini* var. *oncaea**.

*A question mark indicates that the parasite species was identified tentatively by the authors*.

*1, Summarized by Chatton (1920); 2, Sewell (1951); 1/2, Reported by Chatton (1920) and parasite subsequently identified by Sewell (1951); 3, Ianora et al. (1990); 4/1, Reported by Apstein (1911) and parasites subsequently identified by Chatton (1920); 5, Pasternak et al. (1984); 6, Øresland (1991); 7, Skovgaard (2005); 8, Skovgaard and Salomonsen (2009); 9, Drits and Semenova (1985); 10, Alves-de-Souza et al. (2011)*.

*Species names were validated according to Razouls et al. (2005–2012)*.

## Materials and Methods

### New observations on *Blastodinium* spp. hosts, occurrence, and morphology

Unpublished information on new hosts for *Blastodinium* spp. in the Atlantic Ocean (including two undescribed species) originate from the field work described in Skovgaard and Salomonsen (2009). Hitherto unpublished scanning electron microscopy (SEM) observations on *Blastodinium* sp. dinospores were made as part of the study reported by Skovgaard et al. (2007). Most samples for acquisition of new DNA sequences were collected as part of already published studies (Skovgaard and Saiz, 2006; Skovgaard et al., 2007; Skovgaard and Salomonsen, 2009; Alves-de-Souza et al., 2011). Specimens isolates with the prefix “VIL” in Figure 7 are from Villefranche-sur-mer, France. Following samples were collected the 2 of September 2009 at the site “La marinière”: VIL4, VIL57, and VIL59 from *Farranula rostrata*; VIL15, VIL50, and VIL51 from *Corycaeus* sp.; VIL7 from *Triconia* sp.; and VIL8 from *Corycaeus* cf. *ovalis*. VIL49 from *F. rostrata* was collected the 3 of September 2009 at “Le plateau.” VIL61 from *Triconia* sp. was collected the 1 of March 2010 at the B site (43°41′10N 7°18′94E). VIL44 from *Corycaeus* sp. was collected the 9 September 2009 at “La marinière.” Isolates with the prefix “GA” were collected in the North Atlantic Ocean.

### Phylogenetic analyses

*Blastodinium* spp. were dissected from their host and the DNA extracted as described by Alves-de-Souza et al. (2011). Primers for SSU and PCR conditions are also explained in this reference. Primers used to amplify the ITS region were 5′-GTCGCTCCTACCGATTGAGT-3′ (name ITS-CER-F) in forward and 5′-CAGACAGGCATGTCACCTTC-3′ (name ITS-CER-R) in reverse. PCR conditions were similar to that explained for the SSU. Both SSU and ITS1, 5.8S, and ITS2 amplicons were cloned and sequenced as following the procedure by Alves-de-Souza et al. (2011), and consensus sequences were deduced from the analysis of three different clones. SSU and ITS alignments were obtained using the online version of MAFFT^1^. ITS alignment was secondarily manually optimized using secondary structures using models described by Gottschling and Plötner (2004). Non-informative sites were removed using Gblocks^2^. A Bayesian phylogenetic tree was constructed with MrBayes v3.2 (Huelsenbeck and Ronquist, 2001) using a GTR substitution model with gamma-distributed rate variation across sites (GTR + I) as suggested as the best-fit model in MrModelstest v2.3 (Nylander, 2004). Four simultaneous Monte Carlo Markov chains were run from random trees for a total of 1,000,000 generations in two parallel runs. A tree was sampled every 100 generations, and a total of 2,500 trees were discarded as ‘burn-in’ upon checking for stationarity by examination the log-likelihood curves over generations, and posterior probabilities were calculated in MrBayes. A consensus tree (50% majority rule) was constructed from the post-burn-in trees and posterior probabilities were calculated in MrBayes.

### Transmission electron microscopy

For transmission electron microscopy (TEM) the intact copepods, *Faranulla rostrata*, with parasites were fixed by two methods: (1) 1.5 ml of 2.5% glutaraldehyde on 0.05 M cacodylate buffer (pH 7.4) diluted from 0.2 M on marine water were mixed with 0.5 ml of 4% OsO_4_ and added to the sample for 35 min in the dark. Thus, the final concentrations of glutaraldehyde and OsO_4_ were 1.9 and 1% correspondingly. (2) 0.5 ml of 2.5% glutaraldehyde on 0.05 M cacodylate buffer (pH 7.4) diluted from 0.2 M on marine water were mixed with 0.5 ml of 4% OsO_4_ and added to the sample for 40 min in the dark (final concentrations of glutaraldehyde and OsO_4_ were 1 and 2% correspondingly). The dehydration with alcohol series from 30 to 70% followed without rinsing. All steps of fixation and the dehydration were on ice. The material was kept in 70% alcohol in the freezer (−20°C) for a week before the consecutive dehydration and embedding in Epon. The ultrathin sections were prepared using a ultra-microtome Leica ultracut UCT (Leica Microsystems, Germany), stained with uranyl acetate and lead citrate, and viewed in a JEOL JEM 1400 transmission electron microscope (JEOL Ltd., Japan) at 80 kV, equipped with digital camera Orius SC1000 (Gatan Inc., USA).

### Photosynthesis

Photosynthetic rates, P, were measured by the modified single cell technique as described by Skovgaard et al. (2000). Copepods were collected in the NW Mediterranean Sea in November, 2003 (Skovgaard and Saiz, 2006) and used for experiments within the same day. All handling and incubation was done at a temperature corresponding to that of the site of collection (16.2°C). Prior to incubation, copepods (*Oncaea* spp.) were gently picked out individually and rinsed in 0.2-μm filtered, freshly collected seawater. For incubation, copepods were transferred to 20 ml capacity glass scintillation vials containing 2 ml 0.2-μm-filtered seawater. Each vial contained two copepods infected with *Blastodinium* sp. (presumably *B. mangini*). A NaH^14^CO_3_ stock solution was added (specific activity = 100 μCi ml^−1^, Carbon 14 Centralen, DHI-Group, Denmark), resulting in a specific activity of approximately 0.9 μCi ml^−1^. Vials were then incubated for 4 h in triplicates at irradiances of 50, 150, 250, and 350 μmol photons m^−2^ s^−1^ plus a triplicate that was incubated in the dark. After incubation, specific radioactivity of the medium was checked after incubation by transferring 100 μl incubation water from each vial to new vials containing 200 μl NaOH. The amount of fixed ^14^C was measured in the remaining 1.9 ml, which received 2.0 ml of 10% glacial acetic acid in methanol to remove all inorganic C. Vials were dried overnight at 60°C whereupon residues were re-dissolved in 2 ml distilled water and 10 ml of Packard Insta-Gel Plus scintillation cocktail (PerkinElmer, USA) were added to all vials. Finally, new caps (Packard poly screw caps, PerkinElmer) were mounted and activities were determined with a Packard 1500 Tri-Carb liquid scintillation analyzer (PerkinElmer). Calculations of photosynthetic rates, P, were based on the equation given by Parsons et al. (1984).

## Results and Discussion

### Life cycle stages and their morphology

#### Life cycle

The complete life cycle of *Blastodinium* has not been demonstrated definitively, but the morphology of distinct stages of the parasite cycle has been described. According to Chatton (1920), the infection cycle of *Blastodinium* starts with the ingestion of a dinospore by a copepod and, instead of becoming digested, the dinospore grows in size and develops into a trophocyte, which is the earliest parasitic phase that has been identified. In young trophocytes that have recently infected their host, an anterior beak may sometimes be present (Figure 1B).

**Figure 2:**
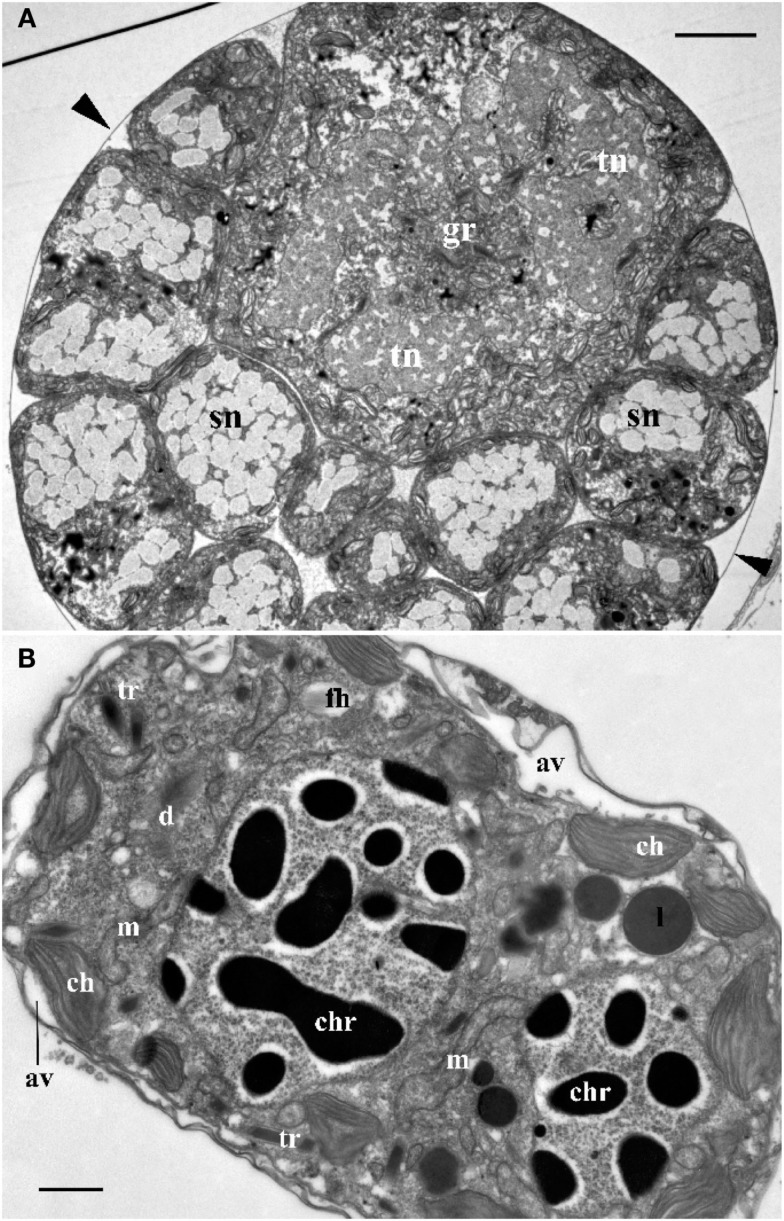
**(A)** Cross section through the central part of *Blastodinium* cf. *mangini* (from *Farranula rostrata*) showing a big trophocyte with several layers of sporocytes covered by common a cuticle (arrowheads). gr, golgi region; sn, nucleus of sporocyte; tn, nucleus of trophocyte. Scale bar = 5 μm. **(B)** Ultrastructure of mature *Blastodinium* cf. *navicula* sporocyte (from *F. rostrata*). av, amphiesmal vesicle (alveolus); ch, chloroplast; chr, chromosome; cu, cuticle; d, dictyosome; fh, vesicle with future flagellar hairs; l, lipid droplet; m, mitochondrion; tr, trichocyst. Scale bar = 1 μm.

**Figure 3:**
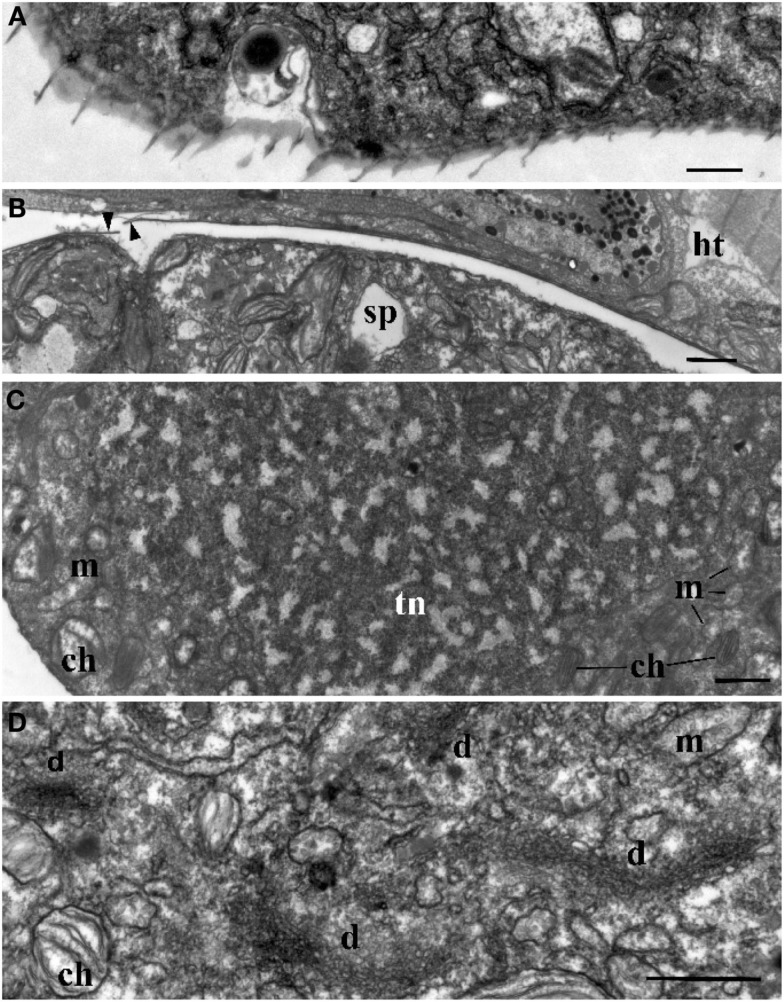
**Ultrastructure of *Blastodinium***. **(A)** Cell covering of *Blastodinium* cf. *mangini* (from *Farranula rostrata*) in crest region. **(B)** Cuticle of trophont attached tightly to the gut tissue of the host (ht); arrowheads show broken cuticle on the left. *Blastodinium* cf. *mangini* (from *F. rostrata*). **(C)** Portion of trophocyte of *Blastodinium* cf. *navicula* sporocyte (from *F. rostrata*) with huge nucleus (tn) containing decondensed chromosomes (light zones) and granular material (possibly ribosomal subunits), small chloroplasts (ch) and mitochondria (m). **(D)** Golgi region of trophocyte of *B*. cf. *mangini* (from *F*. *rostrata*) with prominent dictyosomes (d), chloroplasts (ch), and mitochondria (m). Scale bar = 1 μm.

**Figure 4:**
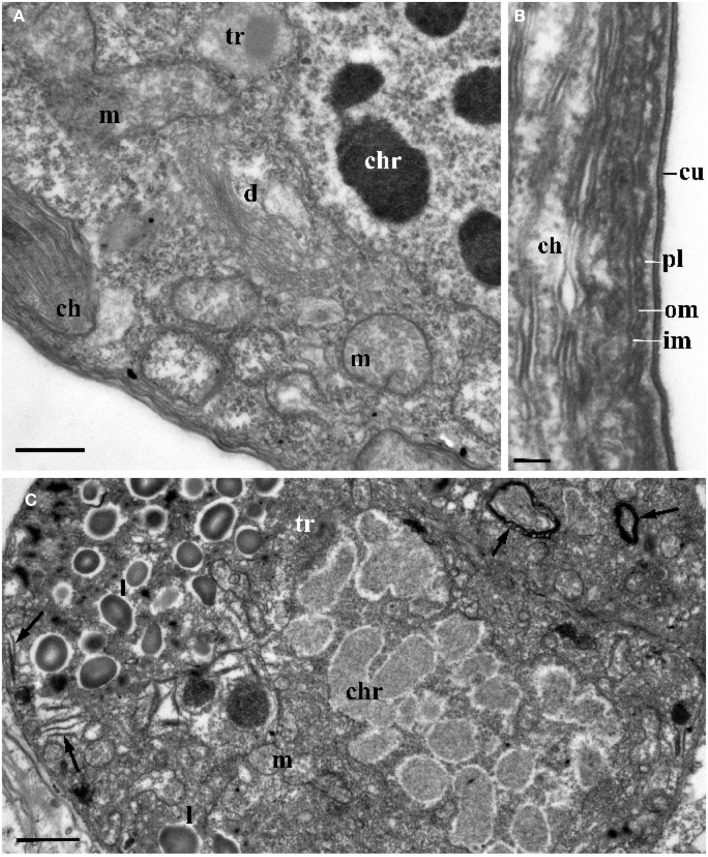
**The ultrastructure of *Blastodinium* sporocyte**. **(A)** Portion of nucleus and cytoplasm of *Blastodinium* cf. *navicula* sporocyte (from *F. rostrata*). **(B)** Structure of coverings of *Blastodinium* cf. *navicula* sporocyte (from *F. rostrata*). **(C)** Colorless representative of *Blastodinium* (*Blastodinium* cf. *hyalinum* from *Paracalanus parvus*). ch, chloroplast; chr, chromosome; cu, cuticle; d, dictyosome; im, inner and outer (om) membrane of alveoli; l, lipid droplets; m, mitochondrion; pl, plasma membrane; tr, maturing trichocyst. Arrows show reduced presumed plastids. **(A)** Scale bar = 2.5 μm. **(B)** Scale bar = 0.1 μm. **(C)** Scale bar = 1 μm.

**Figure 5:**
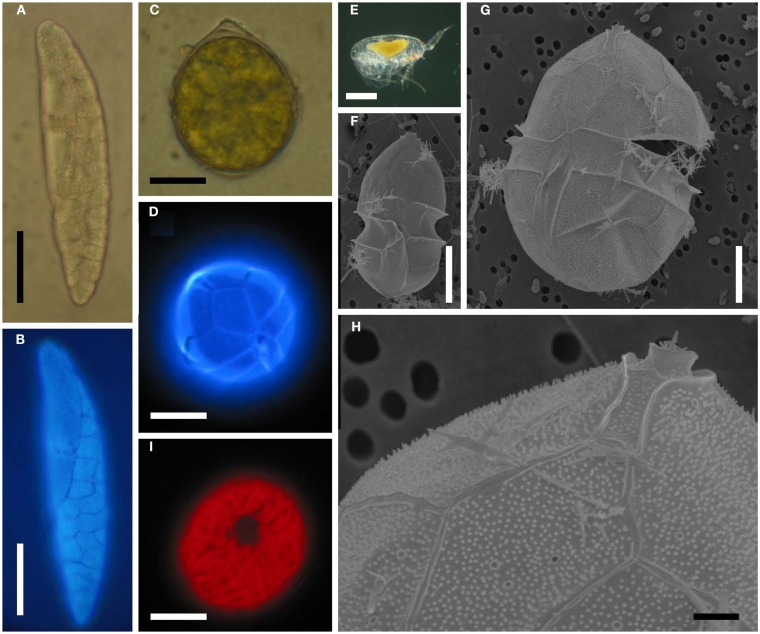
***Blastodinium* spp. (A)** *B. mangini* from *Oncaea* sp. **(B)** Same as **(A)** but stained with Calcofluor White. **(C)** *B. oviforme* dinospore from *O. similis*. **(D)** Same as **(C)** but stained with Calcuflour White. **(E)** *Clausocalanus* sp. infected with *Blastodinium* sp. **(F)** *B. contortum* dinospore, SEM. **(F)** *B. contortum* dinospore, SEM. **(G,H)** Dinospore from the parasite in **(E)**, SEM. **(I)** *Blastodinium* sp. dinospore showing autofluorescence of chloroplasts viewed in epifluorescent light. **(A,B)** Scale bar = 50 μm. **(C,D,I)** Scale bars = 10 μm. **(E)** Scale bar = 200 μm. **(F,G)** Scale bars = 5 μm. **(H)** Scale bar = 1 μm.

Following infection, the trophocyte produces the characteristic large multicellular structure (Figure 1), which corresponds to the parasite undergoing palisporogenetic divisions. Thus, the trophocyte divides into a secondary trophocyte and a gonocyte contained within a common cuticle (Figure 1D). This external cuticle of the sporogenetic individual was described by Chatton (1920) to be formed by the delamination of the mother trophocyte membrane (Figures 2–4). In comparison with the unitary membrane, this cuticle is much thicker, about 15–20 nm (Figure 4B) vs. the thickness of 7–8 nm of the unitary membrane. The cuticle has a three-layered structure: an electron dense inner, a translucent middle, and a comparatively dense outer layer (Figure 4B). None of these three layers has a typical membrane structure. Thus, the cuticle can be considered an extracellular envelope. When stained with Calcofluor White (which stains dinoflagellate thecal plates; Fritz and Triemer, 1985), it is the covering of the underlying cells that is stained rather than the cuticle (Figures 5A,B). After the initial division of the trophocyte, the produced gonocyte will divide into a large number of sporocytes still retained within the external cuticle, resulting in a large multicellular individual (Chatton, 1920; Figures 1 and 5A,B). In some cases (to some extent species dependent) the secondary trophocyte will divide into a tertiary trophocyte, and a new gonocyte will then produce a second layer of sporocytes (Figure 1F). This process may be repeated and result in several layers of sporocytes. Chatton (1920) referred to these conditions as mono-, di-, or polyblastic, dependent on how many layers of sporocytes were surrounding the trophocyte. In some species, the trophocyte is not completely embedded by sporocytes, leaving a “naked” area, a hilum (Figures 1E and 2A), where the trophocyte is visible and in direct contact with the cuticle. Sporulating individuals generally measure up to several hundreds of μm in length and are often detected coincidentally inside the transparent copepod’s gut thanks to their size and the brownish to greenish color caused by the presence of chloroplasts. The rupture of the cuticle leads to the release of sporocytes into the water through the copepod anus.

The surface of the parasite cuticle is at times ornamented with two helical rows of minute spinules along the body (Figure 1C). These “helicoidal crests” can be difficult to discern in the light microscopy, but are readily seen in SEM (Skovgaard and Salomonsen, 2009) and in ultrathin sections (Figure 3A). They are formed by papillae located on the trophocyte (see Figure XXVII, p. 132, in Chatton, 1920). The crests may also be visualized by hematoxylin-staining (Coats et al., 2008). The crests are not one continuous helix, but formed by two distinct sections. This reflects the fact that the trophozoite is composed of “Siamese twins,” each of them having their own nucleus (Chatton, 1920). This dualism is also conserved in sporocytes (Figure 2B). *Blastodinium* spp. do not possess a peduncle or other holdfast organelles (Fensome et al., 1993), but one may speculate that the anterior beak of young trophocytes and/or the spinules of the helicoidal crests may play a role in anchoring the parasite to the copepod gut lining. In addition, sections made on infected copepods show that sporulating *Blastodinium* appear attached to the gut epithelium, and the outer covering has a tight contact with epithelium (Figure 3B).

Some *Blastodinium* species are gregarious, i.e., several individuals of the same parasite are present in one host individual. Such gregarious parasites are thought be the result of a division of the trophocyte by binary fission into two daughter trophocytes (either before or during sporogenesis). Chatton (1920) used the term “endogenous multiplication” for this type of division compared to the sporogenesis that was referred as “exogenous multiplication” or “palisporogenesis.” During endogenous multiplication the two daughter trophocytes are each surrounding by a new cuticle, and they will eventually produce independent, but synchronous, generations of sporocytes. Rupture of the original cuticle will then release two daughter parasites of approximately similar size. Conversely, the infection with one *Blastodinium* species appears to prevent succeeding infections (Chatton, 1920). Multiple individuals in a single host are, thus, typically of approximately similar developmental stage. However, this is not always the case, and Sewell (1951) consequently suggested that successive infections of a single host may also be possible. The final proof of multiple infections would be the presence of two different *Blastodinium* species in a single host, but this has never been observed.

#### The dinospore

Sporocytes released through the copepods anus are initially non-motile. After few hours, these bi-nucleated cells acquire flagella and divide into four uni-nucleate dinospores (Chatton, 1920; Soyer, 1971; Figure 5C). These dinospores are thecate with plates that are clearly visible when stained with Calcofluor White (Figure 5D) as well as in SEM (Figures 5F–H). The pattern and arrangement of thecal plates on *Blastodinium* dinospores is typical for Peridiniales. A pattern of pores encircled by each a single ring gives some resemblance to the peridinoid dinoflagellate genus *Pentapharsodinium* (Skovgaard et al., 2007) on which thecal plates are ornamented with one or two concentric rings. With respect of the thecal tabulation pattern, no distinct differences have been found between the morphology of the species *B. navicula*, *B. contortum*, and *B. galatheanum* (Skovgaard et al., 2007; Skovgaard and Salomonsen, 2009). However, variation in the morphology of *Blastodinium* dinospores does occur. The cell depicted in Figure 5G is considerably larger than the typical dinospore (Figure 5F). Even though the plate tabulation pattern is similar in both types of dinospores, the larger dinospore has a distinctive theca possessing a dense coverage of papillae (Figures 5G,H). These unusual dinospores were collected after having been expelled from *Blastodinium* sp. hosted by a single specimen of *Clausocalanus* sp. (Figure 5E) and they were motile at the time of fixation. Thus the aberrant morphology of these dinospores cannot presently be explained, but it is probable that they represent a species yet not described.

#### Infection and transmission

While it appears logical that *Blastodinium* infects its host through oral transmission by a dinospore, the means of infection has never been proved experimentally. Likewise, the mechanism by which the infective dinospore is subsequently able to avoid digestion and remain in the gut of its host is currently unknown. Attempts to transmit *Blastodinium* experimentally from infected copepods to uninfected individuals have been unsuccessful (Chatton, 1920; Skovgaard, 2005). It has been suggested that adult copepods are not infected, but that infection takes place in the juvenile stages (Chatton, 1920; Alves-de-Souza et al., 2011). This theory might explain the failure of transmission experiments, since these have concentrated on late copepodite stages and adult copepods.

### Ultrastructural modifications during sporogenesis

The *Blastodinium* trophocyte has an aberrant morphology compared to typical dinoflagellates, which is plausibly a result of its parasitic life style. Following successive sporogenetic generations, cells are gradually re-acquiring typical features of free-living dinoflagellates. These morphological transformations can be observed in a single polyblastic individual, since such individual will have different layers of sporocytes of different age (Soyer, 1970, 1971).

#### Nucleus

Ultrastructure of the nucleus during mitotic divisions was studied in detail by Soyer (1971). According to that report and Figures 2–4, *Blastodinium* has a dinokaryotic nucleus at all stages with the nuclear envelope remaining intact during mitosis and chromosomes staying attached to the inner membrane. Invaginations of the nuclear envelope with cytoplasmic microtubules inside are also frequently observed, demonstrating typical dinomitosis. However, although the trophocyte nucleus has dinokaryotic chromosomes (lacking histones), these are decondensed with a large amount of granular contents (ribosomal subunits) around (Figures 2A and 3C). Progressive condensation of chromosomes takes place during sporogenesis (Soyer, 1971). The first sporocyte layers have nuclei with less nucleoplasm and much more condensed chromosomes. They will remain in such condition during several sporogenetic divisions (Figures 2A and 4A,C). The most condensed chromosomes appear in the mature sporocytes (Figure 2B) and this chromosome compaction is concomitant to the reduction of nuclear size.

#### Chloroplasts

Plastids of the trophocyte are rather small, often with light stroma and few thylacoids (like etioplastids in plants; Soyer, 1970; Figures 2A and 3C,D). However, they are fully reactivated during the course of sporogenesis and in mature sporocytes the plastids are well developed and located at the cell periphery (Figure 2B). Pyrenoids are present in later stage only (Figures 2B and 4A). The colorless species *B. hyalinum* seems to possess remnants of chloroplasts, but these are very rare and appear to be highly degenerated (Soyer, 1970; Figure 4C).

#### Trichocysts

*Blastodinium* has typical dinoflagellate trichocysts that are very rare, if present at all, in the trophocyte (Figure 3). Some premature stages of trichocyst development are found in immature sporocytes (Figure 4), and many well-developed trichocysts are present in mature, binucleate sporocytes (Figure 2B).

#### Golgi apparatus

Soyer (1970) also reported additional transformations in the Golgi apparatus and mitochondria along the sporogenetic process. These observations are not confirmed here in sections of *B. mangini*, but Figures 2 and 3D show that the Golgi apparatus was extremely large in the trophocyte, composed of several huge dictyosomes, some of them up to 5 μm in length. It occupies a big region between the two nuclei of the trophocyte (Chatton, 1920; Figures 2 and 3D). Both the nuclear structure and the Golgi structure reveal the intense metabolic activity of the trophocyte stage. In addition, mitochondria were well developed at all stages of *Blastodinium* proliferation having typical dinoflagellate tubular cristae (Figures 3 and 4).

#### Cell covering

Membrane structures surrounding the trophocyte and sporocytes are of similar appearance, being covered by three membranes corresponding to the typical dinoflagellate amphiesma (Figure 4). The alveoli are flat with electron translucent contents. The outer membranes of the alveoli attach tightly to the plasma membrane. However, this amphiesma becomes more elaborate in mature sporocytes, with broader alveoli (Figure 2B). In conclusion, a mature sporocyte with two nuclei has very condensed chromosomes, developed amphiesma, mature trichocysts, and prominent chloroplasts (Figure 2B), which seem to represent typical features of naked immature dinospores.

### Taxonomy

#### Taxonomic position of the genus

Since the discovery of *Blastodinium*, it has been recognized that these organisms exhibit features that separate them from the bulk of the dinoflagellates of the class Dinophyceae. *Blastodinium* has thus been appointed as the type genus of a separate class, the Blastodiniphyceae (Fensome et al., 1993), comprising the single order Blastodiniales. Blastodiniphyceae was synonymized with Haplozooidea and placed in the superclass Hemidinia by Cavalier-Smith (1993). The main character distinguishing the class from the Dinophyceae has been a parasitic life mode and the presence of histones in larger trophic cells and the absence of such in the smaller swarmers (dinospores), i.e., a temporary dinokaryon (Fensome et al., 1993). Recent findings that *Blastodinium* dinospores are thecate with a thecal plate tabulation pattern corresponding to that of Peridinian dinoflagellates (Skovgaard et al., 2007) suggest a closer relationship with the Dinophyceae and this is also supported by molecular phylogeny.

#### Species distinction

The main characters for species discrimination within the genus *Blastodinium* are based upon morphological distinctions of the parasitic stage (Chatton, 1920), such as size, the location of the trophocyte, coloration, number of sporocyte layers, the presence of a hilum, and the existence of helicoidal crests. Another important character is whether the parasites are solitary or gregarious in their hosts. Among the gregarious species, also the number of parasites in each host is given taxonomic importance. Gregarious species typically have 2, 3, or 4 parasites in each host, but some species, such as *B. spinulosum*, are often more numerous; more than 10 is not unusual and up to 23 parasites have been found in a single *B. spinulosum* in *Paracalanus parvus* (Chatton, 1920). Based on these morphological characters, Chatton (1920) arranged *Blastodinium* spp. in three groups, the *spinulosum* group, the *contortum* group (in which the two species described by Sewell also fit), and the *mangini* group (Table 1). The main characteristics of the *spinulosum* group are that the parasites are curved and shaped like a small boat with a rounded anterior pole and a pointed posterior pole, the trophocyte is located in the anterior part, and the parasite body is traversed by a groove and one or more helicoidal crest(s). The *contortum* group is characterized by the parasite body being twisted in early developmental stages, they have no helicoidal crest, no groove, and they are usually solitary in their host. In the *mangini* group, both poles of the parasite are rounded and individuals are nearly straight. Finally, the host species, or the range of host species, is of taxonomic importance.

There are currently 13 accepted species of *Blastodinium* (Table 1), of which the majority were discovered in copepods from the Mediterranean Sea early in the twentieth century (Chatton, 1906, 1908, 1911, 1912, 1920). Two taxa were originally described as varieties (*B. crassum* var. *inornatum* and *B. contortum* var. *hyalinum*; Chatton, 1920) but these have subsequently been generally accepted as independent species, i.e., *B. crassum* and *B. hyalinum*. A couple of species were afterward found in the Arabian Sea and described by Sewell (1951). Since then only a single new species has been identified and described, namely *B. galatheanum* (Skovgaard and Salomonsen, 2009). *B. hyalinum* is the only species that was explicitly described as being colorless (Chatton, 1911). However, the two species named by Sewell (1951), *B. apsteini* and *B. chattoni*, were noted as having closest similarity to *B. hyalinum*, so even though the pigmentation was not mentioned in the description of these two species, one must assume that they were considered to be colorless.

#### Unrecognized morphological diversity

**Figure 6:**
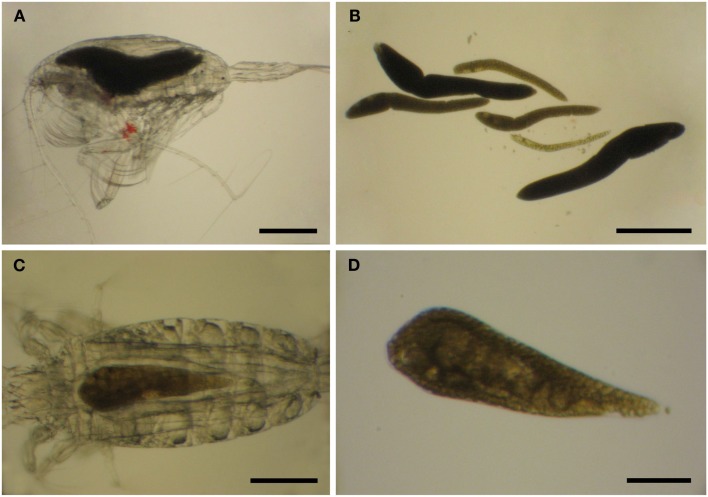
***Blastodinium* sp. in *Euchaeta* sp. from the Atlantic Ocean. (A,C)** Parasites inside their hosts. **(B)** Six parasites of an unidentifiable species from the host in **(A)**. **(C)** A single unidentifiable parasite from the host in **(C)**. **(A,B)** Scale bars = 500 μm. **(C)** Scale bar = 200 μm. **(D)** Scale bar = 100 μm.

Limited work has been done on the taxonomy of *Blastodinium* since the work by Chatton and Sewell, and studies of *Blastodinium* outside European waters are still few. It is, therefore, possible that the diversity within this genus is not yet fully elucidated, and indeed several morphotypes have been observed which cannot be assigned to any known species (Table 1). A study on *Blastodinium* in the Mediterranean Sea revealed several specimens with a morphology that did not match any described species (Alves-de-Souza et al., 2011). The isolate BOUM7 in that study was not only morphologically different from any known *Blastodinium* species; it was also genetically distinct from other members of the genus. Furthermore, during a recent 2-weeks cruise in warm, oligotrophic waters of the central Atlantic Ocean from the Azores Islands to the Southern coast of West Africa, a new *Blastodinium* species was found and described (Skovgaard and Salomonsen, 2009) and a couple of specimens of each two other unknown *Blastodinium* specimen were observed (Figure 6). One of these was a large species (>1 mm long) with six conspicuously colored parasites in a single host individual, *Euchaeta* sp. (Figures 6A,B); the other one a solitary likewise colored specimen in the same host species (Figures 6C,D). The ease by which these unknown members of *Blastodinium* were found reinforces perceptions that the morphological diversity of the genus is presently being underestimated. In fact, scientists tend not to assign species names to individual organisms when studying *Blastodinium* spp. (Pasternak et al., 1984; Ianora et al., 1990; Øresland, 1991), which probably reflects the high morphological variation within the genus resulting in many morphotypes that appear intermediate between recognized species.

### Phylogeny

#### Phylogenetic position of the genus

The temporary dinokaryon has been considered the phylogenetically important character distinguishing the Blastodiniphyceae from the typical dinoflagellates, the Dinophyceae (Saunders et al., 1997), and the Blastodiniphyceae have been considered evolutionary basal to the Dinophyceae. The importance of the temporary dinokaryon as a taxonomic feature has, however, been questioned (Fensome et al., 1993) and its phylogenetic value may also be limited. Furthermore, molecular phylogenetic studies have shown that species originally classified in the Blastodiniphyceae are polyphyletic and have placed several blastodinian dinoflagellate species well within the Dinophyceae (Saldarriaga et al., 2004). Hence, differences in nuclear structure are of dubious phylogenetic significance.

Traditional dinoflagellate morphology-based taxonomy does not always conform to modern taxonomy in which molecular phylogeny is taken into account (Fensome et al., 1999; Saldarriaga et al., 2004) and the phylogeny of dinoflagellates has been emended considerably after molecular phylogeny has been widely incorporated (e.g., Daugbjerg et al., 2000). This also applies to the blastodinian dinoflagellates. While *Blastodinium* has shown affinity to the Peridiniales in molecular phylogenic analyses (Skovgaard et al., 2007), other blastodinian species branch elsewhere within the Dinophyceae (Saldarriaga et al., 2004). According to molecular phylogenetics, Blastodiniphyceae, and Blastodiniales thus have no validity, a fact that may not be surprising when considering the peridinian morphology of the dinospores. However, a formal revision of the taxonomic position of *Blastodinium* has yet to be made and such revision may need to await a more general revision of the Peridiniales and related dinoflagellates.

Even though emerging SSU rDNA sequence data confirms a close taxonomic relation between individual *Blastodinium* species, these are not always monophyletic in phylogenetic analysis (Skovgaard et al., 2007; Alves-de-Souza et al., 2011). A couple of recent analyses have, on the other hand, shown *Blastodinium* to be monophyletic, but the support for this is consistently low (Coats et al., 2008; Skovgaard and Salomonsen, 2009). All these reports have, nevertheless, agreed that *Blastodinium* belong phylogenetically to the typical, dinophycean dinoflagellates.

#### Intrageneric phylogeny

**Figure 7:**
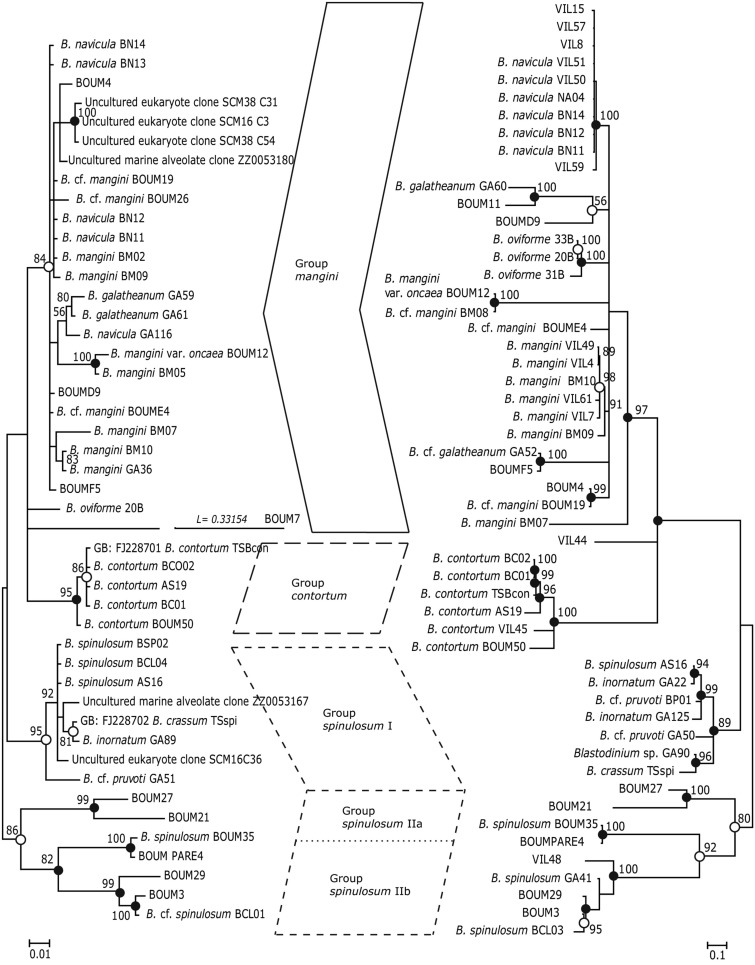
**Phylogenetic trees based on Bayesian analyses of alignments comprising SSU rDNA sequences (left) and ITS1, 5.8S rDNA, and ITS2 sequences (right) of *Blastodinium* spp**. Numbers above nodes are Maximum Likelihood bootstrap values; only values above 50% are shown. Filled circles at nodes denote that the clade had Bayesian posterior probabilities (PP) of 1.00; open circles denote PP of 0.95–0.99. PP < 95 are not shown. Labels at branches are species names and/or isolate names. Two sequences are identified by their GenBank accession number; these have the prefix “GB:” GenBank accession numbers for all sequences are given in **Table A1** in Appendix.

**Figure 8:**
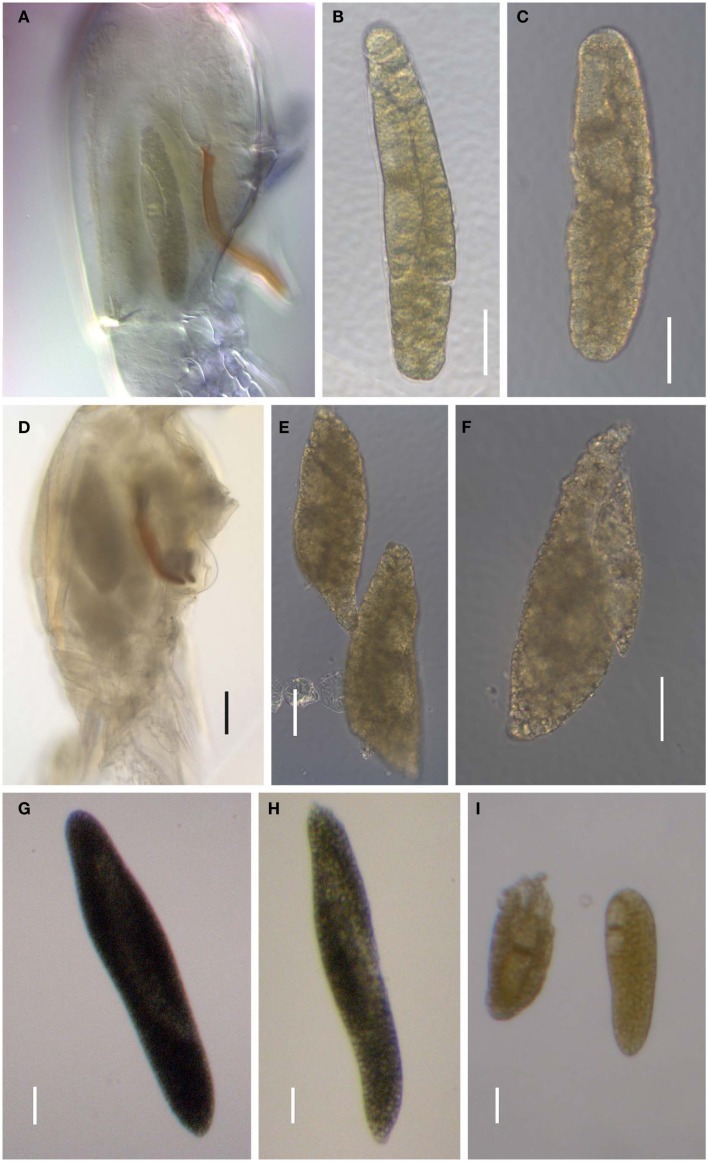
***Blastodinium* spp. (A–C)** *Blastodinium mangini*. **(A)** Three individuals inside *Farranula rostrata* (Villefranche-sur-mer, 2 of September 2009). Monoblastic stage (I-128). **(B)** Individual extracted from *F. rostrata* (Villefranche-sur-mer, 3 of September 2009, VIL49). Monoblastic stage (I-64). **(C)** Individual extracted from *F. rostrata* (Villefranche-sur-mer, 2 of September 2009, VIL52). Monoblastic stage (I-128). **(D–F)** *B. navicula*. **(D)** Four individuals inside *F. rostrata* (Villefranche-sur-mer, 2 of September 2009, VIL50). **(E,F)** Different individuals extracted from the precedent copepod host (VIL50). Monoblastic stage (I-128). **(G)** *Blastodinium* cf. *galatheanum* from *Acartia negligens* (North Atlantic, GA52). **(H)** *B. galatheanum*. from *A. negligens* (North Atlantic, GA60). Scale bar = 50 μm. I. *B. mangini* var. *oncaea*. Two individuals from *Oncaea* sp. (NW Mediterranean Sea, BM05). **(B,C)** Scale bars = 50μm. **(D)** Scale bar = 100 μm. **(E–I)** Scale bars = 50μm. **(G)**

**Figure 9:**
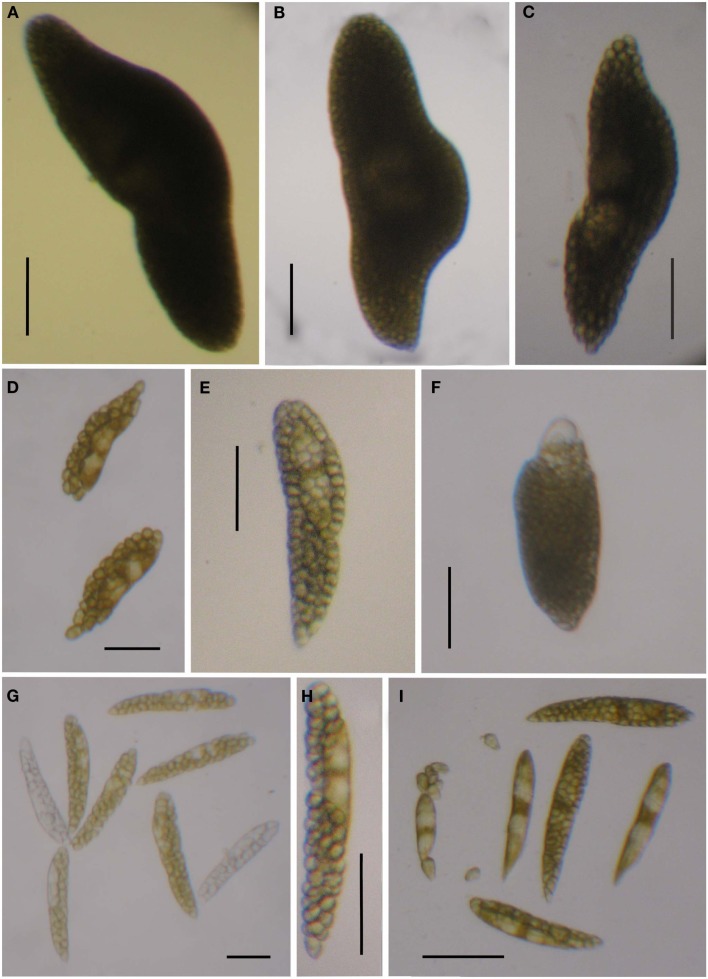
***Blastodinium* spp**. Sequences from samples are used for the phylogenetic analyses in Figure 7. **(A–C)** Solitary individual of *B. inornatum* Group *spinulosum* I from *Clausocalanus* spp. **(A)** GA22. **(B)** GA89. **(C)** GA125. **(D–F)** Gregarious individuals of *Blastodinium* spp. Group *spinulosum* I from *Clausocalanus* spp. **(D)** *Blastodinium* cf. *pruvoti*, BP01. **(E)** *Blastodinium* cf. *pruvoti*, GA50. **(F)** *B. oviforme* from *Oithona similis*. **(G,H)** Gregarious individuals of *B. spinulosum* Group *spinulosum* II. **(G)** *Blastodinium* cf. *spinulosum*, BCL01, from *Clausocalanus* cf. *arcuicornis*. Note two colorless individuals. **(H)** *B. spinulosum*, GA41, from *Paracalanus parvus*. **(I)** *B. spinulosum*, BCL04, Group *spinulosum* I from *Clausocalanus* sp. Scale bars = 100 μm.

Phylogenetic analyses (Maximum Likelihood, ML, and Bayesian inference, BI) of *Blastodinium* spp. based on 18S rDNA and ITS (ITS1, ITS2, and 5.8S rDNA) sequences are presented in Figure 7. Some of the more characteristic specimens sequenced for these analyses are depicted in Figures 8 and 9, including the two newly sequenced species *B. inornatum* and *B. oviforme*. These analyses do not address the potential of lack of monophyly among *Blastodinium* spp., since only *Blastodinium* sequences (including putative *Blastodinium* sequences) are included. Overall, the two data sets (18S rDNA vs. ITS) show consensus with moderate to high support for the *contortum*, *spinulosum*, and *mangini* groups (Figure 7), indicating that gross morphology does reflect molecular phylogeny within the genus. This is despite the fact that the two data sets are based in part on different samples. The *mangini* group has the poorest resolution among the three major groups, and has high support only in the ITS analyses (ML bootstrap value of 97 and BI posterior probability of 1.00). In the *mangini* group, only *B. oviforme* and *B. navicula* ITS sequences branch out as monophyletic. On the contrary, the positions of *B. mangini* (both trees) and *B. galatheanum* (ITS tree) do not agree exactly with the morphology-based classification, and in the 18S phylogeny *B. navicula* is not well resolved. In particular *B. mangini* sequences are very diverse and this harmonizes with the high morphological variation described by Chatton (1920), leading him to erect *B. mangini* var. *oncaea*. The isolate BOUM7 (*Blastodinium* sp.) clusters together with the *mangini* group with a long branch. Indeed, in a previous analysis including also a number of non-*Blastodinium* dinoflagellates, the BOUM7 isolate branched out separately from the other *Blastodinium* clades (Alves-de-Souza et al., 2011).

The consistent high support for the *contortum* group suggests that these sequences may in fact comprise a single species (Figure 7), which is here represented from different host species and different locations (Mediterranean Sea and Gulf of California). The *spinulosum* group, on the contrary, is as a whole well supported, but it is very diverse and the individual clades do not conform entirely with the identification of species as determined through morphological traits. Most typical *B. spinulosum* morphotype sequences do branch in one clade (Group *spinulosum* I), but this clade also encompasses *B. pruvoti*, *B. inornatum*, and *B. crassum* morphotypes (Figure 7). On the other hand, *B. spinulosum* morphotype sequences are also present in the *spinulosum* II clade. One of these *B. spinulosum* morphotypes (BCL01) was morphologically distinct by comprising eight individuals of which two were apparently colorless (Figure 9G).

In conclusion, it is probable that both the *mangini* group and the *spinulosum* group(s) encompass one or more species complexes and that, possibly, a larger species diversity than currently recognized exists within *Blastodinium*. A high genetic diversity within *B. mangini* was to be expected considering the large number of hosts known for this species and the fact that the morphology of *B. mangini* can be quite variable (Chatton, 1920). Unfortunately, sequences from *B. hyalinum*, *B. chattoni*, and *B. apsteini* are currently not available and the phylogenetic position of these colorless species is, thereby, unknown.

### Occurrence

#### Species distribution

**Figure 10:**
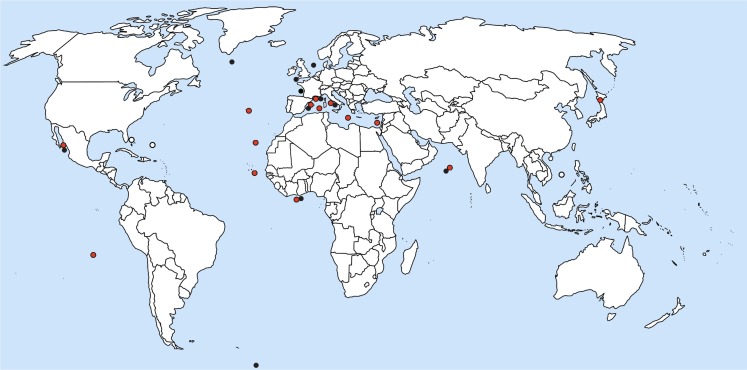
**Location of current reports of *Blastodinium* spp. in marine copepods**. Red circles represent photosynthetic species; black circles are colorless species; and white circles are environmental DNA sequences with high similarity to *Blastodinium*.

**Figure 11:**
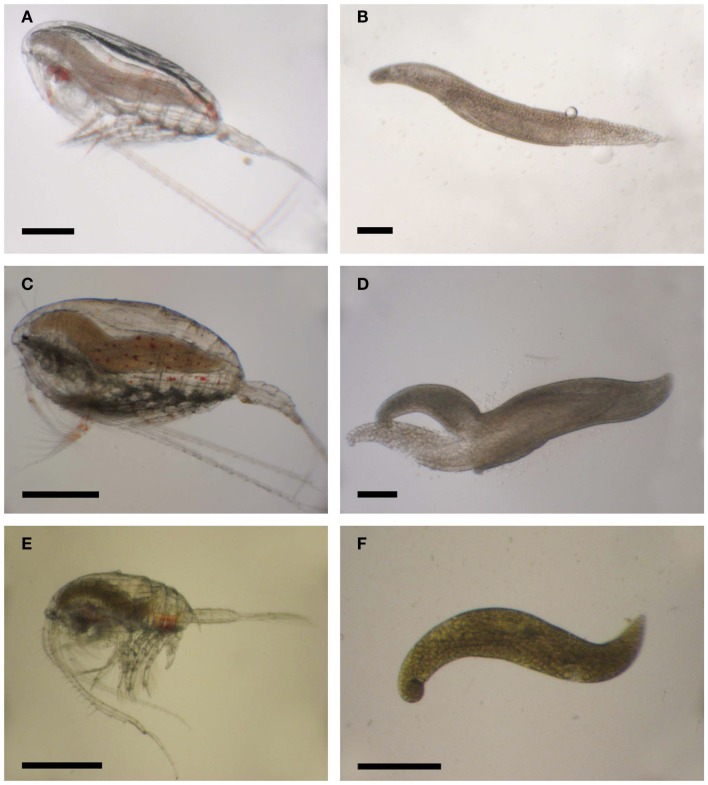
***Blastodinium* spp. in calanoid copepods**. **(A)** *Calanus* sp. from Greenlandic waters with *B. hyalinum*. **(B)** Parasite from **(A)**. **(C)** *Cosmocalanus vulgaris* off the West coast of Africa with *Blastodinium* cf. *chattoni*. **(D)** Parasite from **(C)**. **(E)** *Temora stylifera* from the Central Atlantic Ocean with *Blastodinium* cf. *contortum*. **(F)** Parasite from **(E)**. **(A,C,E)** Scale bars = 500 μm; **(B,D,F)** scale bars = 200 μm.

Reports on *Blastodinium* spp. suggest that the genus occurs world-wide in seawater with close to full strength salinity, i.e., more than approximately 30 ppt. (Figure 10). Most observations on *Blastodinium* have been done in coastal waters, but this pattern is likely to reflect the ease of access to sampling sites rather than the actual distribution of the organisms. Based on the work by Apstein (1911) and subsequent observations, Chatton (1929) noted that *B. hyalinum* was the only species present in the cold temperate North Sea, whereas a row of green (photosynthetic) species existed in warm temperate waters of the Mediterranean Sea. This observation concurs with that of subsequent studies, finding exclusively *B. hyalinum* in cold temperate waters (Lebour, 1925; Jepps, 1937; Vane, 1952). Also the species reported by Øresland (1991) in *Euchaeta antarctica* from Antarctic waters was presumably *B. hyalinum* considering the length of the parasite (2.5–3.5 mm; Øresland, 1991), matching no other known *Blastodinium* species. As further support for *B. hyalinum* being a world-wide species, Figures 11A,B depict *B. hyalinum* in *Calanus* sp. from Greenlandic waters. On the other hand, photosynthetic species of *Blastodinium* are restricted to warm temperate, subtropical, and tropical waters. These waters are often oligotrophic suggesting that the life strategy of *Blastodinium* spp. has adapted to such an environment. One may speculate that a semi-parasitic organism, a “parasitic alga,” as *Blastodinium* will benefit from being able to acquire inorganic nutrient from its host in oligotrophic waters, thereby avoiding potential nutrient limitation. Considering the relatively small number of surveys, it is to be expected that both the geographic range and the host range of *Blastodinium* are broader than now recognized. A further addition to the known geographic range of *Blastodinium* cf. *chattoni* is given in Figures 11C,D, showing this parasite in *Cosmocalanus vulgaris* collected in the Central Atlantic Ocean off the West coast of Africa by Skovgaard and Salomonsen (2009). From the same waters, a rare example of a *Temora stylifera* was also found infected with a *Blastodinium* (Figures 11E,F), in this case a species that was identified as an early developmental stage of *B. contortum*.

#### Occurrence of dinospores

Free-swimming *Blastodinium* cells (dinospores) are rarely encountered in the marine plankton. This may be because dinospores are short-lived and therefore less likely to be detected, but it is also possible that *Blastodinium* dinospores are overlooked due to their superficial resemblance with free-living, non-parasitic peridinoid dinoflagellates. Alves-de-Souza et al. (2011) found numerous dinospores in the Mediterranean Sea using DNA-probes and proposed that some of the “small thecate dinoflagellates” often observed in mixed plankton samples may in fact be *Blastodinium* dinospores. Also, SSU rDNA sequences from Sargasso Sea nanoplankton show high similarity with *Blastodinium* spp. and are most likely to originate from *Blastodinium* dinospores (Skovgaard and Salomonsen, 2009). The same may apply for two ITS1 sequences from the South China Sea (GenBank accession numbers GU941876 and GU942050). Little is known about the vertical distribution of *Blastodinium* spp. dinospores, but Alves-de-Souza et al. (2011) found maximum densities of dinospores at or just below the deep chlorophyll maximum in the Mediterranean Sea (approximately 100 m), i.e., in an environment where photosynthetic growth is unlikely to be of any significance. Occurrence of *Blastodinium* spp. is seasonal (Chatton, 1920; Skovgaard and Saiz, 2006) suggesting that the life cycle may contain a dormant stage. Indeed, Chatton (1920) mentioned the presence of cysts, but their fate and function remain unknown.

#### Host-specificity

Most *Blastodinium* species infest several copepod host species. This was originally realized on the basis of *Blastodinium* morphology (Chatton, 1920; Sewell, 1951) and it is corroborated by recent ITS sequences. For example sequences of isolate VIL61 from *Triconia* sp. were identical to VIL4 and VIL49 (Figure 8B) from *F. rostrata*. However, there is a clear distinction between species that infect calanoid copepods and those that infect cyclopoid copepods. This pattern was noted already by Chatton (1920, p. 236) and has been corroborated in subsequent investigations (Table 1). Also, there is a large variation in the number of host species a single *Blastodinium* species is able to infect, even though this observation may depend on how commonly the parasite species is observed. It is interesting to note that several common copepod species, such as *Oithona nana* and *T. stylifera*, are rarely infected with *Blastodinium* spp. in mixed plankton communities in which several other copepod species have high infection prevalence (Skovgaard and Saiz, 2006).

### Ecology

#### Photosynthetic growth

**Figure 12:**
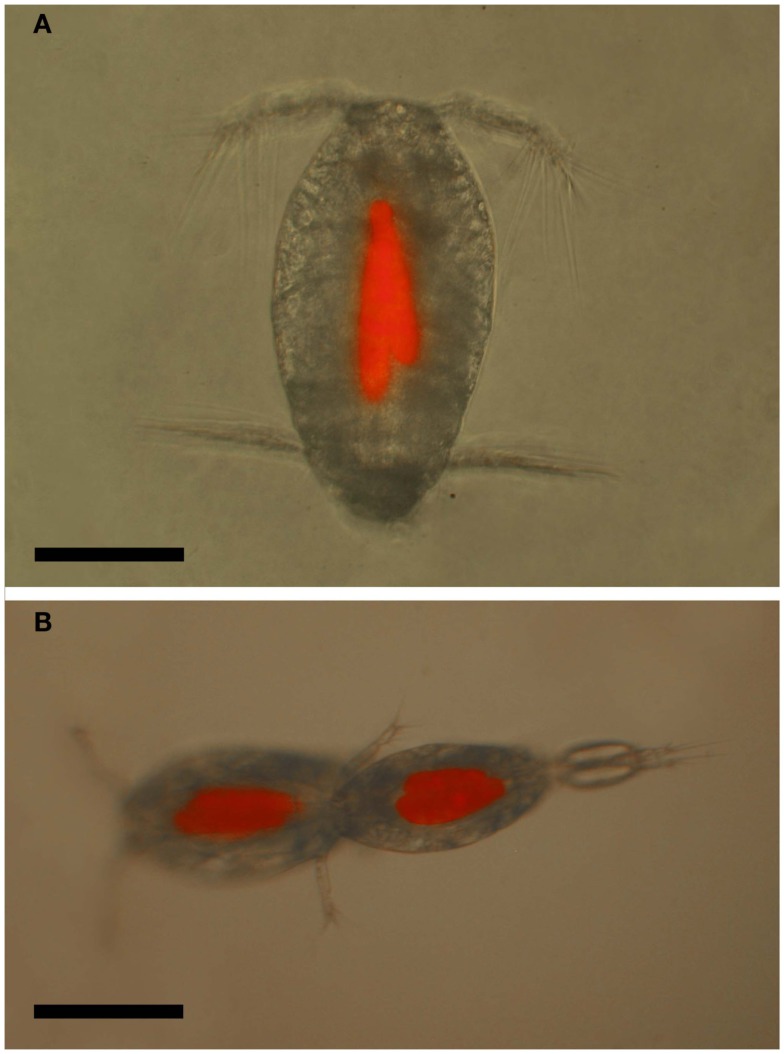
***Oncaea* cf. *scottodicarloi* with *Blastodinium mangini* in epifluorescent illumination showing autofluorescence of parasite chloroplasts**. **(A)** Female with two parasites. **(B)** Copulation male (right) and female (left), both infected with 2–3 parasites. Scale bars = 100 μm.

The gut of planktonic copepods has been shown to be a suitable environment for photosynthesis (Epp and Lewis, 1981) and since most *Blastodinium* species possess well-developed chloroplasts it is natural to assume that they are capable of photosynthesis. Pasternak et al. (1984) demonstrated that this is in fact the case and made the crude estimate that *Blastodinium* sp. was able to fulfill approximately half of its energy needs through photosynthesis, implying that the other half must somehow be obtained from the host. To further test this, the photosynthetic rate of *Blastodinium* cf. *mangini* hosted by *Oncaea* spp. (Figure 12A) was determined in November in the NW Mediterranean Sea (Table 2). The photosynthetic rate, P, was up to 826 pg C parasite^−1^ h^−1^ corresponding to a P per volume of the parasite of 1.6 ng C cm^−3^ h^−1^. At the lower irradiance of 50–150 μE m^−2^ s^−1^, P/vol was 0.6–0.9 ng C cm^−3^ h^−1^. The magnitude of P may be put in perspective by comparing with P of mixotrophic free-living dinoflagellates with a known relative contribution of photosynthesis for cell growth: P/vol of *Gyrodinium resplendens* was 2.5 ng C cm^−3^ h^−1^ at 75 μE m^−2^ s^−1^ (Skovgaard, 2000), and P/vol of *Fragilidium subglobosum* was 2.8 ng C cm^−3^ h^−1^ at 150 μE m^−2^ s^−1^ (Skovgaard et al., 2000). Hence, the photosynthetic activity of *Blastodinium* cf. *mangini* is less than half of that of the two free-living, mixotrophic dinoflagellates. *F. subglobosum* acquired only 10% of its C needs through photosynthesis under the conditions at which P was measured (Skovgaard et al., 2000), and *G. resplendens* acquired approximately 16% of its C demand through photosynthesis. Assuming that all factors are equal, *Blastodinium* cf. *mangini* should then acquire an even smaller fraction of its C needs through photosynthesis. According to this crude approximation, the estimate that *Blastodinium* sp. should fulfill approximately half of its energy needs through photosynthesis (Pasternak et al., 1984) is not unrealistic, but may be a comparatively high estimate. The existence of *Blastodinium* species with apparently non-functional chloroplasts (i.e., the colorless species) gives good reason to believe that *Blastodinium* spp. are able to obtain a substantial part of their energy needs heterotrophically through organic substances acquired from the host.

**Table 2:** **Photosynthetic rates, P, of *Blastodinium* sp. inside *Oncaea* sp. at four different irradiances**.

Irradiance (μmol photons m^−2^ s^−1^)	P^a^ (pg C parasite^−1^ h^−1^)	SE (*n* = 3)	P/vol^b^ (ng C μm^−3^ h^−1^)
50	400	–	0.6
150	509	6	0.9
250	574	26	1.1
350	826	13	1.6

*^a^Each replicate contained two copepods hosting each two parasites, i.e., measured P was four times that reported here*.

*^b^P per volume of parasite. Volume calculated according to a prolate ellipsoid. Average dimension of parasites used: L = 200 μm, W = 70 μm*.

*Blastodinium* dinospores also contains chloroplasts (Figure 5I), suggesting a potential for photosynthetic growth. This could hypothetically be an advantage for the dispersal of the parasite, since dinospores would be able to stay alive for longer time outside their host. However, in a culture experiment, *B. mangini* dinospores did not survive for longer than a week, regardless whether they were incubated in light or in the dark (Skovgaard, 2005). Yet, in the optimum environment one would still expect a beneficial effect of chloroplasts for the survival time of dinospores and this could potentially increase the probability of finding a new suitable host. It is also possible, on the other hand, that the main photosynthetic activity occurs in the sporocytes, thereby supporting growth of the parasite inside the host. According to this hypothesis, the chloroplasts in dinospores may be a vestige from the preceding photosynthetic stage.

### Effects on the host

#### Growth and fitness

Infection with *Blastodinium* spp. generally leads to a reduced size of the infected host (Chatton, 1920; Sewell, 1951; Alves-de-Souza et al., 2011). This may be caused by food limitation induced by the parasite physically blocking the alimentary tract, but it is also possible that the parasite is utilizing organic matter in the gut, thereby competing with the host for the ingested food. Infected copepods also have a decreased survival as compared to uninfected copepods, which is possibly caused by the same food limitation mechanisms (Skovgaard, 2005). One could speculate that hosting a photosynthetic organism would be advantageous for the host and that the parasite would a beneficial symbiont supplying its host with organic matter. However, a comparison of survival of infected vs. uninfected *Oncaea* spp. incubated both in light and in the dark did not reveal any difference and thus did not give any support for *Blastodinium* being beneficial to its host (Skovgaard, 2005).

#### Castration

*Blastodinium* spp. is able to induce castration of infected female copepods (Chatton, 1920; Sewell, 1951; Skovgaard, 2005) and gonads are usually not fully developed in infected organisms (Chatton, 1920). Incubation experiments have demonstrated that infected copepods usually do not deposit eggs (Ianora et al., 1990; Skovgaard, 2005) and, even though infected females have been found carrying eggs at a few occasions (Vane, 1952; Skovgaard, 2005), hatching of eggs produced by an infected female copepod has never been witnessed. On the other hand, histological and ultrastructural examinations of *Blastodinium*-infected copepods, have shown ovaries and reproductive diverticulae to be normal in size and shape and oogonal development appeared comparable to uninfected individuals (Ianora et al., 1990). It thus appears that an infection with *Blastodinium* spp. does not necessarily destruct the gonad anatomy, as is the case with the more detrimental parasite *Syndinium turbo* (Ianora et al., 1990). The infection does, however, lead to physiological castration, which is probably induced by the parasite “stealing” energy from its host and/or by the mechanical pressure exerted by the parasite on the reproductive organs. Some parasitic castrators are able to modify the scheme by which the host allocates energy, thereby shunting resources from reproduction to growth (Hall et al., 2007). This mechanism is not likely to be of importance for *Blastodinium* spp. given the fact that infected copepods are smaller than healthy specimens.

#### Female vs. male infection

In general, only adult females and juveniles are infected with *Blastodinium* spp. Chatton (1920) found a large number of female and male copepodites to be infected, but did not find a single infected, adult male. He ascribed this to a failure of infected male copepodites of molting to the adult stage. Later studies have shown *Oncaea* spp. to be an exception to be above rule, since male *Oncaea* spp. are frequently infected with *B. mangini* (Sewell, 1951; Skovgaard, 2005) and so are males of species within Corycaeidae (Alves-de-Souza et al., 2011). Infections in adult males of other species are extremely rare: Sewell (1951) found a single adult *Nanocalanus minor* male to be infected with *B. contortum*. This represents the only of two examples of *Blastodinium* infection in an adult calanoid male, the other example being a male *Acartia clausi* observed by Ianora et al. (1990).

The prevailing hypothesis predicts that *Blastodinium* spp. are transmitted through oral infection. Such entry of infection would explain the lack of infection in adult males, since males of many copepod species are short-lived and do not have the capability to feed, in particular among the Clausocalanoidea (Ohtsuka and Huys, 2001). If infected, juvenile males are unable to molt to the adult stage; this would efficiently prevent the existence of infected males. *Oncaea* spp. are, on the other hand, among those species in which males do feed (Ohtsuka et al., 1996). Hence, the pattern of infection in males supports the hypothesis of oral transmission of infection. There are no records of behavioral changes in copepods due to infection with *Blastodinium* spp. On the contrary, males do mate with infected females (Cattley, 1948) and, in the case of *Oncaea* spp., both infected males and females have been observed mating, and even two infected individuals will mate with each other (Skovgaard, 2005; Figure 12B).

#### Sex alternation

Copepods parasitized by *Blastodinium* spp. have been observed to be morphologically intersexual (Jepps, 1937; Cattley, 1948; Sewell, 1951; Ianora et al., 1987) and this has been interpreted as parasite-induced sex reversal (Cattley, 1948). However, the proof that parasitism is a main causal factor for intersexuality and sex reversal in copepods is non-existent, since sex alternation is common among copepods – also among non-parasitized individuals (Shields, 1994). Furthermore, intersex is caused by several factors other than parasitism, e.g., mortality, differential longevity, and environmental factors such food as limitation (Gusmão and McKinnon, 2009). Nevertheless, it does appear that intersexuality is more common among copepods parasitized by *Blastodinium* spp. than among non-parasitized copepods (Sewell, 1951 and parasitism should, therefore, be considered among the environmental factors causing intersexes in copepods (Gusmão and McKinnon, 2009).

#### Prevalence

Most current knowledge on the occurrence, diversity, and prevalence of *Blastodinium* spp. is from the Mediterranean Sea and this is also the only site from where seasonal data are available. These data show marked seasonal variations and demonstrate that prevalence and diversity of the genus are highest from mid-summer through autumn; *Blastodinium* spp. are rare in late winter and during spring (Chatton, 1920; Skovgaard and Saiz, 2006). Quantitative investigations on *Blastodinium* spp. from the Mediterranean Sea and the Arabian Sea indicate that it is not unusual to find peaks among adult females of some copepod species with 20% or more infected (Table 3), but a prevalence below 5–10% is more common (Chatton, 1920; Sewell, 1951; Skovgaard and Saiz, 2006) and some copepod species are infected only to a very low extent. Limited quantitative data is available from cold temperate waters. Vane (1952) found overall infection rates of 3.7 and 3.8% for adult and stage V females of *Calanus finmarchicus* and *Pseudocalanus elongatus*, respectively, from the Continuous Plankton Recorder samples taken from the North Sea (Hardy, 1941). Maximum infection rates were noted to be as high as 66 and 60%, respectively, but sample sizes were, unfortunately, not specified. Other studies have found low numbers of parasites in the North Sea and adjacent waters (Apstein, 1911; Jepps, 1937).

**Table 3:** **Prevalence of *Blastodinium* spp. in different copepod hosts**.

Parasite	Host	Prevalence(%)	Location	Reference
*Blastodinium crassum*	*Paracalanus parvus*	1.5	W Mediterranean Sea	1
*B. contortum*	Subeucalanus subtenuis*	6–12	SE Pacific Ocean	2
	*P. parvus*	0–3.9	W Mediterranean Sea	6
*B. hyalinum*	*Calanus finmarchicus*	3.7–66	North Sea	3
	*Pseudocalanus elongatus*	3.8–60	North Sea	3
	*P. elongatus*	0.3–20.5	North Sea	4
	*Paraeuchaeta antarctica*^+^	6.6	Weddel Sea	5
*B. mangini*	*Oncaea* cf. *scottodicarloi*	0–17	W Mediterranean Sea	6
	*Farranula rostrata*	10	W Mediterranean Sea	1
*B. navicula*	*Corycaeus giesbrechti*	20–30	W Mediterranean Sea	1
*B. oviforme*	*Oithona* spp.	0–3	W Mediterranean Sea	6
*B. spinulosum*	*P. parvus*	0.4–0.7	W Mediterranean Sea	1
	*Clausocalanus* spp.	0.9	W Mediterranean Sea	1
*Blastodium* spp.	*P. parvus*	0.4	W Mediterranean Sea	7
	Calanoida	33^#^	E Mediterranean Sea	8
	Corycaeidae	51^#^	E Mediterranean Sea	8
	Oithonidae	<2^#^	E Mediterranean Sea	8
	Oncaeidae	<2^#^	E Mediterranean Sea	8

*, The parasite was presumably *B. contortum*; ^+^, the parasite was presumable *B. hyalinum*; ^#^, based on DNA-stain screening.

*1, Chatton (1920); 2, Pasternak et al. (1984); 3, Vane (1952); 4, Cattley (1948); 5, Øresland (1991); 6, Skovgaard and Saiz (2006); 7, Ianora et al. (1987); 8, Alves-de-Souza et al. (2011)*.

Given the diversity and host-specificity of *Blastodinium* spp., a considerable effort is needed to assess the existence of all *Blastodinium* species in their respective potential hosts, and as a consequence, prevalence is often known only from sporadic investigations and often mainly for copepod species that tend to have highest degree of infection. This could imply that the general prevalence is overestimated. However, the opposite is more likely to be the case, because early developmental stages of parasites are almost certainly systematically overlooked and, furthermore, parasites are typically looked for in preserved samples in which the parasite have lost its color and become less conspicuous. The detection of endoparasites can be facilitated by fixation and storage of samples under conditions that preserve the color of the parasite (Skovgaard and Saiz, 2006), but even under optimum conditions early parasite stages are in risk of being overlooked. A solution to this problem may be to apply cell or DNA stains. Unspecific DNA dyes allow for a rapid detection of the DNA-rich nuclei of *Blastodinium* cells. A recent study used DNA-staining to detect infections and found relatively high infection prevalences; 51 and 33% for Corycaeidae and Calanoidae, respectively (Alves-de-Souza et al., 2011). It may also be possible to stain *Blastodinium* spp. differentially by use other standard staining techniques, such as hematoxylin. The practical feasibility of such staining techniques for quantitative samples is, however, in need for future validation.

#### Effect on host populations

Little is known about the impact of *Blastodinium* parasitism on natural copepod populations. The direct adverse effect of *Blastodinium* on fitness and survival of the infected host has been documented (Skovgaard, 2005), but has not been accounted for in field investigations. A single study has explored the adverse effect of *Blastodinium* spp. on the population of two copepod species. In this case it was estimated that impairment of the reproductive rate of *Oncaea* cf. *scottodicarloi* females infected with *B. mangini* was 0.05–0.16 day^–1^ and for *P. parvus* females infected with *B. contortum* the impairment was up to 0.03 day^–1^ (Skovgaard and Saiz, 2006). The magnitude of reproductive impairment was concluded to be comparable in effect on host populations to that of sources of predator-induced mortality. *Oncaea* spp. males are also infected to a considerable extent (Sewell, 1951; Skovgaard, 2005, Figure 10B) and infected male and female copepods appear to copulate actively (Figure 12B), but possibly copulating with an infected male is never successful, thereby reducing a healthy female’s probability of reproducing successfully with a fertile male. Thus, in addition to the parasite-induced castration of females, copulations in which any of the mates are infected will reduce the overall mating success rate, and infection of males will lead to an enforced reduction in this mating success. This mechanism may have a considerable effect on the recruitment success in individual copepod populations.

## Concluding Remarks

Based on the above compilation of historical and recent data combined with new data presented here, it can be concluded that the genus *Blastodinium* is both morphologically and genetically very diverse. It is, thus, certain that the current number of described species does not reflect the actual diversity of the genus: several *Blastodinium* morphotypes and gene sequences cannot be assigned to any known species. Furthermore, DNA sequences from some of these aberrant morphotypes are highly divergent (such as the BOUM7 isolate; Alves-de-Souza et al., 2011). Therefore, the genus *Blastodinium* is not always monophyletic in phylogenetic analyses encompassing the entire Dinokaryota (Alves-de-Souza et al., 2011) and in those analyses that show a monophyletic origin of the genus, the support is typically negligible (Coats et al., 2008; Skovgaard and Salomonsen, 2009). Finally, as shown in the present study (Figures 7–9) there is only partial agreement between the morphological distinction of *Blastodinium* species and their separation in molecular phylogenetic analyses. This suggests that cryptic speciation exists within the genus and that morphological characters may be insufficient for the separation of individual species.

Even though most *Blastodinium* species contain functional plastids, there is no evidence of any beneficial effects for a copepod hosting a *Blastodinium*. As shown here, chloroplasts are not fully developed in the trophocyte and, thereby, seem to primarily play a role in the growth of sporocytes. However, as discussed above, *Blastodinium* individuals are conceivably highly dependent on organic matter acquired from the host and this energy flow may be a major cause of the harmful effects recorded on infected copepods. The effects of a *Blastodinium* infection is not detrimental to individual copepod hosts, but infection does lead to decreased fitness and physiological castration, which have the potential to significantly influence the affected copepod populations.

An accumulating number of investigations of the occurrence of *Blastodinium* spp. in the World’s oceans strengthen earlier presumptions that photosynthetic species are common but restricted to warm oligotrophic oceans. On the other hand, the colorless species, at least *B. hyalinum*, appear to occur world-wide. Yet, most zooplankton studies do not report on the presence of *Blastodinium* because parasites are generally not considered in standard zooplankton counts and they are easily overlooked by the untrained eye. Nevertheless, the prevalence and effects of *Blastodinium* infections justify that these organisms should ideally be taken into account when assessing zooplankton productivity processes and an important task in future research will be to develop techniques that facilitate registration of parasites in routine zooplankton investigations.

## Conflict of Interest Statement

The authors declare that the research was conducted in the absence of any commercial or financial relationships that could be construed as a potential conflict of interest.

## Footnotes

^1^http://mafft.cbrc.jp/alignment/server/

^2^http://molevol.cmima.csic.es/castresana/Gblocks_server.html

## Appendix

**Table A1:** **GenBank accession numbers for the sequences used in Figure 7**.

Species	Isolate	Host	Number of *Blastodinium* ind. per host	Sampling site	SSU	ITS
***B. contortum***						
*B. contortum*	AS19	*Paracalanus parvus*	1	NW Med. Sea	DQ317536	JX473668
*B. contortum*	BC01	*Clausocalanus arcuicornis*	1	NW Med. Sea	DQ317537	JX473669
*B. contortum*	TSBcon	*Paracalanus parvus* cf.	1	Gulf of California	FJ228701	FJ228701
*B. contortum*	BOUM50	Calanoida	1	Med. Sea	JN257680	JX473687
*B. contortum*	BCO02	*Nanocalanus minor*	1	NW Med. Sea	JX473667	JX473667
*B. contortum*	VIL45	*Paracalanus parvus*	1	Med. Sea	—	JX473688
***B. crassum***						
*B. crassum*	TSspi	*Paracalanus parvus* cf.	1	Gulf of California	FJ228702	FJ228702
***B. galatheanum***						
*B. galatheanum*	GA59	*Acartia negligens*	1	Atlantic Ocean	FJ541187	—
*B. galatheanum*	GA61	*A. negligens*	1	Atlantic Ocean	FJ541188	—
*B. galatheanum*	GA60	*Acartia* cf. *danae*	1	Atlantic Ocean	—	JX473670
*B*. cf. *galatheanum*	GA52	*A. negligens*	1	Atlantic Ocean	—	JX473671
***B. inornatum***						
*B. inornatum*	GA89	*Clausocalanus* sp.	1	Atlantic Ocean	HQ226069	—
*B. inornatum*	GA22	*Clausocalanus* sp.	1	Atlantic Ocean	—	JX473672
*B. inornatum*	GA125	Calanoida	1	Atlantic Ocean	—	JX473673
***B. mangini***						
*B. mangini*	BM02	*Oncaea* sp.	3	NW Med. Sea	JX473655	—
*B. mangini*	BM05	*Oncaea* sp.	2	NW Med. Sea	JX473656	—
*B. mangini*	BM07	*Oncaea* sp.	2	NW Med. Sea	JX473657	JX473674
*B*. cf. *mangini*	BM08	*Oncaea* sp.	2	NW Med. Sea	—	JX473675
*B. mangini*	BM09	*Oncaea* sp.	nd	NW Med. Sea	JX473664	JX473664
*B. mangini*	BM10	*Oncaea* sp.	nd	NW Med. Sea	JX473658	JX473676
*B. mangini*	GA36	*Oncaea* sp.	3	Atlantic Ocean	JX473659	—
*B. mangini*	VIL49	*Farranula rostrata*	3	Med. Sea	—	JX473689
*B. mangini*	VIL4	*Farranula rostrata*	3	Med. Sea	—	JX473690
*B. mangini*	VIL61	*Triconia* sp.	2	Med. Sea	—	JX473691
*B. mangini*	VIL7	*Triconia* sp.	2	Med. Sea	—	JX473692
*B*. cf. *mangini*	BOUM19	*Farranula* cf. *rostrata*	3	Med. Sea	JN257674	JX473701
*B*. cf. *mangini*	BOUME4	*Farranula* cf. *rostrata*	nd	Med. Sea	JN257677	JX473702
*B*. cf. *mangini*	BOUM26	Not determined	nd	Med. Sea	JN257676	—
***B. navicula***						
*B. navicula*	BN11	*Corycaeus giesbrechti*	nd	NW Med. Sea	DQ317538	JX473677
*B. navicula*	BN12	*C. giesbrechti*	nd	NW Med. Sea	JX473665	JX473665
*B. navicula*	BN13	*C. giesbrechti*	nd	NW Med. Sea	JX473660	—
*B. navicula*	BN14	*C. giesbrechti*	nd	NW Med. Sea	JX473661	JX473678
*B. navicula*	GA116	*Corycaeus furcifer*	8	Atlantic Ocean	JX473662	—
*B. navicula*	NA04	*C. giesbrechti*	4	NW Med. Sea	—	JX473679
*B. navicula*	VIL50	*Corycaeus* sp.	4	Med. Sea	—	JX473693
*B. navicula*	VIL51	*Corycaeus* sp.	7	Med. Sea	—	JX473694
*Blastodinium* sp.	VIL15	*Corycaeus* sp.	1	Med. Sea	—	JX473695
*Blastodinium* sp.	VIL57	*Farranula rostrata*	5	Med. Sea	—	JX473696
*Blastodinium* sp.	VIL8	*C*. cf. *giesbrechti*	5	Med. Sea	—	JX473697
*Blastodinium* sp.	VIL59	*Farranula rostrata*	4	Med. Sea	—	JX473698
***B. oviforme***						
*B. oviforme*	20B	*Oithona* sp.	1	NW Med. Sea	JX473666	JX473666
*B. oviforme*	31B	*O. similis*	1	NW Med. Sea	—	JX473680
*B. oviforme*	33B	*O. similis*	1	NW Med. Sea	—	JX473681
***B. pruvoti***						
*B. pruvoti*	GA50	*Clausocalanus* sp.	5	Atlantic Ocean	—	JX473682
*B. pruvoti*	GA51	*Clausocalanus* sp.	5	Atlantic Ocean	FJ541189	—
*B. pruvoti*	BP01	*Clausocalanus* sp.	2	NW Med. Sea	—	JX473683
***B. spinulosum***						
*B. spinulosum*	AS16	*P. parvus*	nd	NW Med. Sea	HQ226070	JX473700
*B. spinulosum*	BCL04	*Clausocalanus* sp.	10	NW Med. Sea	HQ226071	—
*B. spinulosum*	BSP02	*Clausocalanus* sp.	20	NW Med. Sea	HQ226072	—
*B. spinulosum*	BOUM35	Not determined	1	Med. Sea	JN257671	JX473699
*B*. cf. *spinulosum*	BCL01	*Clausocalanus* sp.	9	NW Med. Sea	JX473663	—
*B. spinulosum*	GA41	*P. parvus*	13	Atlantic Ocean	—	JX473684
*B. spinulosum*	BCL03	*Clausocalanus* sp.	14	NW Med. Sea	—	JX473685
***BLASTODINIUM* SP**.						
*Blastodinium* sp.	SCM16C3	Env. sequence		Sargasso Sea	AY664985	—
*Blastodinium* sp.	SCM38C54	Env. sequence		Sargasso Sea	AY664986	—
*Blastodinium* sp.	SCM38C31	Env. sequence		Sargasso Sea	AY664984	—
*Blastodinium* sp.	SCM16C36	Env. sequence		Sargasso Sea	AY664982	—
*Blastodinium* sp.	ZZ0053180	Env. sequence		Florida Straits	EU818565	—
*Blastodinium* sp.	ZZ0053167	Env. sequence		Florida Straits	EU818553	—
*Blastodinium* sp.	BOUMD9	*Farranula* cf. *rostrata*	2	Med. Sea	JN257679	JX473703
*Blastodinium* sp.	BOUM29	Not determined	nd	Med. Sea	JN257672	JX473704
*Blastodinium* sp.	BOUM3	Not determined	nd	Med. Sea	JN257673	JX473705
*Blastodinium* sp.	BOUM4	*Farranula rostrata*	nd	Med. Sea	JN257678	JX473706
*Blastodinium* sp.	BOUM7	Not determined	nd	Med. Sea	JN257681	—
*Blastodinium* sp.	BOUM21	Not determined	nd	Med. Sea	JN257667	JX473707
*Blastodinium* sp.	BOUM27	Not determined	nd	Med. Sea	JN257668	JX473708
*Blastodinium* sp.	BOUMF5	*Farranula rostrata*	3	Med. Sea	JN257669	JX473709
*Blastodinium* sp.	BOUM PARE4	*Paracalanus* sp.	11	Med. Sea	JN257670	JX473710
*Blastodinium* sp.	BOUMB12	*Oncaea* sp.	1	Med. Sea	JN257675	JX473711
*Blastodinium* sp.	BOUM11	Not determined	nd	Med. Sea	—	JX473712
*Blastodinium* sp.	VIL44	*Corycaeus* sp.	nd	Med. Sea	—	JX473713
*Blastodinium* sp.	VIL48	*Paracalanus parvus*	2	Med. Sea	—	JX473714
*Blastodinium* sp.	GA90	*Acartia* sp.	1	Atlantic Ocean	—	JX473686
